# Does Coordinated Postpartum Care Influence Costs?

**DOI:** 10.5334/ijic.2487

**Published:** 2017-03-31

**Authors:** Elisabeth Zemp, Andri Signorell, Elisabeth Kurth, Oliver Reich

**Affiliations:** 1 Swiss Tropical and Public Health Institute, Basel, CH; 2 University of Basel, CH; 3 Helsana Group, Department of Health Sciences, Zürich, CH; 4 Zurich University of Applied Sciences, Institute of Midwifery, CH

**Keywords:** Postnatal care, cost analysis, midwifery, home visits, coordinated care, maternal-child health services

## Abstract

**Questions under study:**

To investigate changes to health insurance costs for post-discharge postpartum care after the introduction of a midwife-led coordinated care model.

**Methods:**

The study included mothers and their newborns insured by the Helsana health insurance group in Switzerland and who delivered between January 2012 and May 2013 in the canton of Basel Stadt (BS) (intervention canton). We compared monthly post-discharge costs before the launch of a coordinated postpartum care model (control phase, n = 144) to those after its introduction (intervention phase, n = 92). Costs in the intervention canton were also compared to those in five control cantons without a coordinated postpartum care model (cross-sectional control group: n = 7, 767).

**Results:**

The average monthly post-discharge costs for mothers remained unchanged in the seven months following the introduction of a coordinated postpartum care model, despite a higher use of midwife services (increasing from 72% to 80%). Likewise, monthly costs did not differ between the intervention canton and five control cantons. In multivariate analyses, the ambulatory costs for mothers were not associated with the post-intervention phase. Cross-sectionally, however, they were positively associated with midwifery use. For children, costs in the post-intervention phase were lower in the first month after hospital discharge compared to the pre-intervention phase (difference of –114 CHF [95%CI –202 CHF to –27 CHF]), yet no differences were seen in the cross-sectional comparison.

**Conclusions:**

The introduction of a coordinated postpartum care model was associated with decreased costs for neonates in the first month after hospital discharge. Despite increased midwifery use, costs for mothers remained unchanged.

## Introduction

There is a growing trend towards earlier hospital discharge after birth [1]; a trend driven mainly by policies of cost containment [2]. The health risks and benefits of early discharge of mothers and their newborns and of varying models of follow-up care have been investigated, particularly in the UK, the US and Northern Europe in the 1990s [3,4,5,6]. Whereas adverse neonatal outcomes were reported in large epidemiological studies [7,8], early discharge was shown to be safe in controlled studies with a post-hospital follow-up programme [9]. Some home visiting programmes had a positive impact on breastfeeding success, maternal confidence and reduction of postpartum fatigue and depression [4,10,11]. Only a few studies have addressed economic aspects of reducing the length of postnatal hospital stays and indicate overall cost savings [12,13,14,15]. Cost reductions were observed for women with low risk pregnancies [12] as well as for women after caesarean section [13], and were dependent on the number of midwifery home visits [14]. Care models based on parents’ preferences were the most cost-minimising [15]. A systematic review of cost effectiveness studies of domiciliary visits in the UK and the US reported only a few studies that showed potential for net cost savings through reduced hospital costs, in particular [16]. One cost-effectiveness study in the US examined the impact of longer hospital stays following legislation obliging health insurers to cover a minimum duration of 48 hours after vaginal birth and 96 hours after caesarean section. A one hour increase in the length of stay resulted in a 2.6% decrease in neonatal morbidity and the costs per life year gained were reported to be 19, 800 USD [17]. Bowers et al. explored the consequences of reducing the length of hospital stays for costs and quality of care in the UK, where postnatal length of stay has fallen substantially in the last decade to the point where almost 70% of women remain in the hospital for less than two days [18]. The authors state that reductions in postnatal stays and related costs may be achievable but necessitate enhanced maternity care in the community to maintain quality of care for mothers and babies.

In countries where diagnosis-related group reimbursement (DRG) is in place, care provision and respective costs have shifted from the in-hospital to the ambulatory sector [19,20,21,22,23]. In Switzerland, a 2014 analysis of health insurance data (Helsana Group) showed only weak evidence of a shift (about 1%) of care provision from the acute in-patient to the ambulatory sector [24]. No specific analyses for postpartum care were performed with this database.

Shifting early postnatal care from in-patient to community-based settings requires intensified network building between inpatient and outpatient care providers, which is seen as an intermediate position between full service segregation and full integration [25]. Yet, fragmentation and lack of continuity among maternity care services often hamper adequate follow-up care for mothers and their newborns, even in high-resource countries [26,27,28,29,30]. New forms of inter-professional and inter-organisational collaboration have been developed [31,32] and implemented for patients with chronic diseases [33], however this has rarely been the case in perinatal care [34]. A comparative study in four Canadian health regions showed that the regions with the highest level of inter-organisational collaboration had the most accessible perinatal health services and the most continuous care delivery [28]. Whether coordination of care yields better patient outcomes and reduces health costs is still under discussion [35,36]. For postnatal care, evidence is absent.

In Switzerland, average postnatal hospital stays have more than halved over the last 50 years, from nearly two weeks to 5.7 days in 2012 [37,38]. With the introduction of DRGs in 2012, a further decrease was observed, to three days following spontaneous vaginal birth [39]. While about 60% of the families had organised an independent midwife for follow-up home visits after discharge — reimbursed by mandatory health insurance [40] — 40% did not receive post-discharge midwifery care. The latter may reflect that families were not informed of post-discharge services, or they decided they did not need such services or they could not find a midwife because of a shortage of community-based working midwives [41]. In the Basel region, a midwife-led coordinated care model, Familystart, was launched in 2012 to guarantee access to post-discharge care for all new families and to develop systematic collaboration between inpatient and outpatient postnatal care services. This coordinated care model should cover any unmet needs occurring at the interface between in-hospital care and community-based services, and deliver safe, high quality outpatient care in the postnatal phase [42,43].

What was unclear, however, was whether the model increased or lowered costs covered by the mandatory health insurance. For this reason, a cost analysis was conducted to investigate health insurance costs for the post-discharge phase of deliveries before and after the introduction of Familystart.

## Description of the integrated postnatal care model Familystart

The midwife-led coordinated postpartum care model, Familystart, guarantees postpartum care at home for each mother- child pair discharged from either of the two obstetric clinics in Basel Stadt (the University Women’s Clinic and the Bethesda private clinic). Familystart’s integrated care strategy is based on a needs analysis of new parents [44] and on the theoretical concept developed by D’Amour [32], which operationalises inter-professional collaboration and organisational and interactional factors. An inter-professional experts’ board was established, consisting of hospital and community-based paediatricians, obstetricians and midwives, and a health authorities’ representative. The board is to be consulted for advice and support on professional and health policy issues. A mixed working group of midwives and health visitors developed a guideline on providing collaborative care for new families and organised inter-professional continuing education in postnatal care for nurses, midwives and health visitors in order to strengthen evidence-based standards and to develop shared visions and goals for postnatal care provision. To improve organisational collaboration, the following actions were implemented: a signed contract between hospitals and community-based midwives that guarantees follow-up care organised by the Familystart Network (the first contract between hospitals and a midwife-led network in Switzerland); clarification and streamlining of service transfer procedures from hospital to midwifery home care, to the community-based health visitors (‘Mütter- und Väterberatungsstellen’); a free telephone helpline run by midwives, accessible from 8 am to 8 pm. Furthermore, to actively manage stakeholders, the leaders of the midwives’ network established and maintain a continuous exchange with in- and outpatient postnatal care providers to improve collaboration. Avenues for efficient information exchange between hospitals, community-based midwives and health visitors were developed or optimized. To improve interactional factors of collaboration, inter-professional meetings were scheduled to discuss working approaches, develop mutual trust and strengthen local networking.

## Methods

We used the database of the largest health insurer in Switzerland, the Helsana Group, to recruit the study population. The study included mothers insured by the Helsana Group and who delivered between January 2012 and May 2013 in the canton of Basel Stadt (BS), the intervention canton where Familystart began in November 2012, and their newborn children. We also included mothers and their newborns from five control cantons without a coordinated postpartum care model (Aargau, St. Gall, Solothurn, Thurgau and Zurich). Mothers with a basic compulsory insurance coverage were identified through a claim with a delivery-related DRG starting in this period. Newborns were identified either through the unique family number at Helsana or by combining the mother’s address and the date of birth of the child from the hospital where the mother had delivered.

In a pre-/post design, post-discharge costs covered by the Helsana Group were analysed, first for a 10-month period before the introduction of Familystart and then for a seven-month period after the introduction of Familystart. The shorter period of measurement in the post-intervention phase was due to time restrictions imposed by the research programme funding the study. Based on a hospital code, data could also be analysed separately for the two hospitals participating in Familystart. A subsequent cross-sectional comparison was made between mothers and their newborns delivered in BS and those from the five control cantons for the period 1 January 2012 to 31 May 2013.

For the study period, costs could be retrieved for 97% of all bills relating to mothers. Costs were calculated as the average (and median) monthly post-discharge costs for mothers and for those children also insured with Helsana Group. As the data for costs were skewed, boot strap estimation was used to derive 95% confidence intervals of mean cost differences between the groups [45]. Costs for mothers and children, respectively, were compared before (longitudinal control group) and after (intervention group) the introduction of Familystart and displayed as boxplots up to the sixth month after delivery. For this analysis, an individual perspective was chosen, starting with the day of the woman’s hospital entry. The in-hospital period was assessed as t_0_ and 30-day periods after hospital discharge were labelled as month1, month2, etc. up to the end of the treatment period. In case of overlap of treatment between these monthly periods, costs were assigned proportionally to the duration of the treatment. Of the 236 women having delivered in BS, the exact date of delivery was unavailable for 56, thus they were excluded from the post-delivery cost calculation. Subsequently, monthly costs of the intervention canton (intervention group) were compared to those in the five control cantons (cross-sectional control group). For this analysis, costs were compared per calendar month, from January 2012 to May 2013. Furthermore, the duration of pre- and post-delivery hospital stay was calculated and analysed analogously. DRG codes were grouped into four categories: vaginal delivery without (V) and with complications (Vk) and caesarean section without (C) and with complications (Ck). Additional information was extracted from the Helsana Group database to control for possible confounding factors: mother’s age, insurance type (private/semi-private, basic), amount of the deductible (a flexible amount of out-of-pocket expenses insured persons can chose. The higher the deductible, the lower are insurance fees), length of hospital stay, and use of midwifery service. Being insured in a managed care model was included in the models for ambulatory costs as well.

Multivariable regression analyses were used to determine whether the coordinated care model was associated with an increase or decrease of costs for mothers and children when controlling for other cost determinants. These analyses were conducted for costs for mothers and costs for children in the first month after hospital discharge. A sensitivity analysis was also conducted for costs occurring in first two months after hospital discharge.

All statistical analyses were performed using R version 3.1.0 (2014–04–10) (R Foundation for Statistical Computing, Vienna, Austria) [46,47].

## Results

### Study population

There were 16, 424 women throughout Switzerland insured at the Helsana Group and who had delivered within the study period. Of them, 236 mothers had delivered in BS and 7, 767 had delivered in one of the five control cantons. Overall, 46% of mothers had an uncomplicated vaginal delivery, 19% had a vaginal delivery with complications, 21% had an uncomplicated caesarean section and 14% had a caesarean section with complications. Table 1 shows the characteristics of i) mothers in BS who had delivered after the introduction of Familystart in one of the two participating clinics (intervention group, n = 92), ii) mothers in BS having delivered before the introduction of Familystart (longitudinal control group, n = 144) and iii) mothers from the five control cantons (cross-sectional control group, n = 7, 767). Mothers in the intervention group were slightly older and had a C-section with slightly more frequency than those in the control groups. The average duration of hospital stay after delivery was somewhat shorter in BS, both in the intervention and longitudinal control groups. The use of midwifery services was highest in the intervention group (80% versus 72% in the longitudinal control group in BS and 67% in the cross-sectional control group). There were no differences in the proportion of mothers having private/semiprivate insurance and only around 1% was in a managed care system. The proportion of mothers with a low deductible (< 500 CHF) was highest in the intervention group.

**Table 1:** Characteristics of mothers in the intervention and the two control groups.

	Intervention group^1^		Longitudinal control group in BS/BL^2^		Cross-sectional control group in 5 cantons^3^	

	N	%	N	%	N	%

n	92	100	144	100	7,767	100

Age (mean years)	33.5		32.6^+^		31.7^+^	

Type of delivery^#^:						
Vaginal, uncomplicated	37	40.2	62	43.1	3, 346	43.1
Vaginal, with complications	14	15.2	32	22.2	1, 712	22.2
C-section, uncomplicated	24	26.1	26	18.1	1, 585	20.4
C-section with complications	17	18.5	24	16.7	1, 124	14.5

Mean (median) duration of days of hospital stay (post-delivery)	3.6(3.0)		3.5°(3.0)		4.0°°(4.0)	
Use of service by midwife^§^	74	80.4	104	72.2	5, 197	66.9
Insurance type^#^:						
Private/semi-private	11	12.0	20	13.9	856	11.0
Managed care	1	1.1	2	1.4	32	0.4
Deductible (CHF)^§^:						
< = 500	64	69.6	76	52.8	4, 908	63.2
> 500–2,000	7	7.6	31	21.5	1, 510	19.4
> 2,000–2,500	21	22.8	37	25.7	1, 341	17.3

^1^Mothers delivering in Basel-Stadt (BS) after the introduction of Familystart (after 1 Nov. 2012). ^2^Mothers delivering in BS before the introduction of Familystart (before 1 Nov. 2012). ^3^Mothers delivering in one of the five control cantons (Aargau, St. Gallen, Solothurn, Thurgau, Zürich), between 1 January 2012 and 31 May 2013. ^+^Significantly different from intervention group (Scheffe Test, p = 0.0031). °Significantly different from intervention group (Kruskaal-Wallis rank sum test, p = 0.0030). °°Significantly different from intervention group (Kruskaal-Wallis rank sum test, p = 0.0002). ^§^Significant differences between the three groups (Pearson’s Chi-squared test, p < 0.001). ^#^No significant differences between the groups (Pearson’s Chi-squared test).

### Re-hospitalisations and emergency consultations

There were only a few re-hospitalisations of mothers within the six months following discharge (six in total, two prior to and four after the introduction of Familystart). Re-hospitalisations of children were more frequent, overall 10% were re-hospitalised (24/236), of whom 17/141 (14%) were re-hospitalised before and 7/95 (8%) after the introduction of Familystart (difference not statistically significant).

Of the 16 registered emergency consultations with children, 10 occurred before and six after November 2012 (not statistically significant). Of the 129 emergency consultations with mothers, there was no significant difference before and after the introduction of Familystart (70 versus 59 consultations).

### Cost comparisons

**Figure 1:**
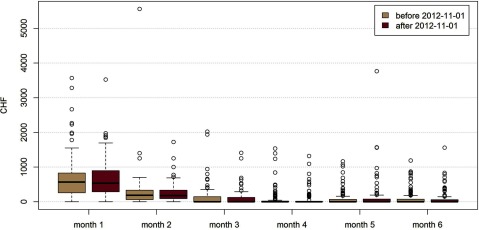
Average monthly costs (in CHF) for post-discharge care for mothers, before (n = 236) and after (n = 95) the introduction of the coordinated care model, Familystart, in Basel.

In the longitudinal comparison, the monthly post-discharge costs for mothers (before and subsequent to the introduction of Familystart in BS) remained unchanged for six months (Figure 1), despite higher costs incurred for the increased use of midwives. Cost differences between BS and the control cantons were minimal (in month 2 and 4), and not consistent in direction.

**Figure 2:**
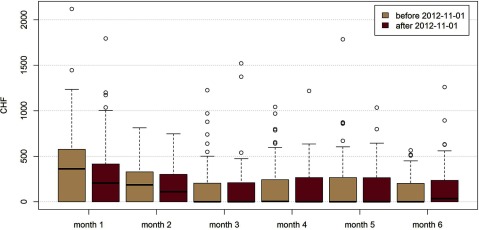
Average monthly costs (in CHF) for post-discharge care for children, before (n = 141) and after (n = 95) the introduction of coordinated care model, Familystart, in Basel.

For children delivered in BS, post-discharge costs during the first month were statistically significantly lower after the introduction of Familystart (Figure 2), by –114 CHF (95%CI –202 CHF to –27 CHF). The proportion of children with post-discharge costs during the first month was lower in the intervention group (60% compared to 73% in the control group, borderline significance). During the subsequent five months, no differences of costs were observed. In the cross-sectional comparison between BS and the control cantons no cost differences were seen for infants.

### Determinants of costs

The longitudinal analysis modelled post-discharge costs for mothers during the first month after delivery, with delivery after the introduction of Familystart as the main determinant of interest (Table 2). In this model, no significant association was observed. Only the use of midwifery services and duration of the post-delivery hospital stay were significantly associated with costs. The type of delivery was not associated with costs. A separate model including post-discharge costs during the first two months after delivery gave a very similar picture (data not shown).

**Table 2:** Determinants of post-discharge costs for mothers during the first postpartum month; a longitudinal comparison of Basel-Stadt pre-intervention (n = 117) and post-intervention (n = 63)*.

	Coefficient Estimate	Std. Error	t value	p value

Intervention group	6.50	91.78	0.07	0.94
Length of hospital stay (post-birth) (per day)	103.99	40.66	2.56	0.01
Use of midwifery service (ref: no use)	481.99	97.37	4.95	0.000
Maternal age (per year)	2.88	9.04	0.32	0.75
Caesarean-section, uncomplicated^1^	–1.20	138.66	–0.01	0.99
Vaginal birth, with complications^1^	–18.26	116.01	–0.16	0.88
Caesarean-section, with complications^1^	13.70	156.81	0.09	0.93
Private/semi-private insurance (ref: mandatory insurance)	21.64	122.95	0.18	0.86
Deductible > 500 CHF (ref.: < 500 CHF)	–0.01	0.05	–0.23	0.82
Managed care (ref.: all other care)	22.75	570.84	0.04	0.97

^1^Reference: vaginal birth without complications. *56 mothers excluded because precise date of delivery was unknown.

The cross-sectional analysis modelled post-discharge costs for mothers in a multivariable regression analysis, with BS (intervention canton) as the main determinant of interest (Table 3). There was no significant association with the place of delivery. Again, the use of midwifery services was associated with higher costs; the incremental costs for midwife support were 432 CHF. This model also showed significant associations between type of delivery and costs (higher costs for complicated vaginal delivery and complicated caesarean section), and with the deductible (lower costs for a deductible of 2, 000 CHF). A small positive effect on costs was also seen for the length of the post-delivery stay. Analogous analyses of potential cost determinants for children during the first month yielded no significant associations (data not shown).

**Table 3:** Determinants of post-discharge costs for mothers; cross-sectional comparison between Basel-Stadt (n = 63) and control group from five cantons* (n = 6191).

	Coefficient Estimate	Std. Error	t value	p value

Intervention group^1^	93.41	61.23	1.53	0.13
Length of hospital stay (post birth) (per day)	12.08	4.70	2.57	0.01
Use of midwifery service (ref: no use)	431.07	13.14	32.81	< 0.001
Maternal age (per year)	1.64	1.25	1.31	0.19
C-section, uncomplicated^2^	28.19	17.23	1.64	0.10
Vaginal, with complications^2^	78.98	16.66	4.74	< 0.001
C-section, with complications^2^	137.97	20.53	6.72	0.000
Private/semi-private insurance (ref: mandatory insurance)	–5.03	19.64	–0.26	0.80
Deductible > 500 CHF (ref.: < 500 CHF)	–0.05	0.01	–6.52	< 0.001
Managed care (ref.: all other care)	–140.23	105.47	–1.33	0.18

^1^Reference: cross-sectional control group (*5 control cantons Aargau, St. Gallen, Solothurn, Thurgau, Zürich). ^2^Reference: vaginal birth without complications.

Further multivariate regression analyses were conducted to investigate possible reasons underlying the different length of hospital stays in BS and in the control cantons (data not shown). In the longitudinal analysis (before/after start of Familystart), duration of hospital stay was not associated with delivery under the integrated care model, only with type of delivery (caesarean section, complicated vaginal delivery) and with supplementary private hospital insurance coverage.

In the cross-sectional analysis, a lower mean duration was associated with delivering in BS compared to delivering in a control canton, and also with a higher deductible. Higher mean length of hospital stay was associated with higher maternal age, the type of delivery (caesarean section, complicated vaginal delivery) and having private/semiprivate insurance coverage.

## Discussion

In our study, average monthly costs for mothers remained unchanged in the seven months after the introduction of a coordinated postpartum care model, despite a higher use of midwifery services, which increased from 72% to 80%. Likewise, costs did not differ between the intervention canton and the five control cantons (average use of midwives 68 %). The longitudinal comparison for the first month after hospital discharge showed lower costs for children after the introduction of Familystart compared to the pre-intervention phase, with a difference of –114 CHF (95%CI –202 to –27) per person, whereas the cross-sectional comparisons with five control cantons showed no differences.

Cost reductions associated with postnatal home care programmes have been reported by previous studies on postnatal care for a low risk population in Switzerland [12] and for women after caesarean section in the US [13]. In a comparative study in Canada, cost reduction due to early discharge and follow-up care at home was dependent on the number of home visits [14]. A retrospective US study analysing only post-discharge costs for neonates found a significant cost reduction when infants received a nursing home visit [48]. Reviews of home visiting programmes showed a cost-reducing effect of home visits for children and families and for elderly patients [16,49] and there is emerging evidence for the cost-effectiveness of midwife-led continuous models of maternity care [50]. Long-term studies on home visiting programmes for disadvantaged families with infants estimate benefit-to-cost ratios around 3:1 to 5:1. In early infancy, cost savings were mainly due to a reduced occurrence of emergency consultations [51].

It is noteworthy that costs for mothers did not increase despite an increase in the use of midwifery services from 72% to 80%. In multivariable analyses, the post-discharge costs for mothers did not differ between the pre- and post-intervention phases. However, in the cross-sectional analysis, they were associated with midwifery use. Health cost savings may have occurred simultaneously with some cost increases associated with the greater use of midwives. Higher quality of care may have been achieved by a range of measures to foster inter-professional collaboration, such as the inter-professional experts’ board, a mixed working group of midwives and health visitors, or the guideline on collaborative care provision and continuing education. The increased access to midwifery care after hospital discharge is in line with previous studies showing an association between the degree of inter-professional collaboration and accessibility of services [52,53]. In both hospitals in the intervention canton (University Women’s Clinic and Bethesda), all mothers were screened in the hospital to determine if they had a midwife for the postpartum period and were systematically informed about and offered home care by Familystart. Some mothers had organised postpartum care prior to the delivery.

The reduction in costs for children may have occurred indirectly as midwives can charge services only to the mother’s bill and not to the child’s. Midwives may have prevented emergency consultations or paediatric consultations for children by informing and reassuring parents. The number of re-hospitalisations was very low, both among mothers and children, and unlikely to account for the observed cost difference for children. The longitudinal comparison of paediatric costs did not reveal any difference. To substantiate the claim that families used paediatric services less frequently after the introduction of Familystart, more data on children would be needed, such as their age at discharge.

Overall costs may vary with the length of hospital stay, thus, determinants of hospital-stay length are of interest. A complicated mode of delivery was expected to be associated with longer hospital stays [17]. Yet, higher age and having private/semiprivate insurance coverage were also predictors for longer hospital stays. Taking private/semiprivate insurance coverage as a proxy variable for higher socio-economic status, this result is in line with previous findings that socio-economically disadvantaged and very young women had shorter postnatal hospital stays [17,54]. This raises the question of whether discharge decisions were really based on medical and needs-oriented criteria. Parity is also known to influence the length of hospital stay, with first-time mothers preferring longer stays [14,17]. This information was not available in our data source. We cannot exclude the possibility that parity caused some confounding if the impact of parity on length of stay was reduced with the introduction of DRGs. A progression towards shorter hospital stays (pre- and post-delivery) was already taking place in the years prior to the introduction of the DRG funding scheme and the establishment of Familystart [38]. Similarly, the use of midwifery services was already relatively high before the introduction of Familystart in the intervention canton (72.2%). This may have reduced the chance to observe a difference in the longitudinal comparison, which only considered the year before and after the introduction of Familystart.

The study has several limitations and strengths worth mentioning. The intervention group was small, limiting the power of detecting differences, for instance, for hospital re-admissions. Also, we cannot exclude some residual confounding by information not assessed in the database, such as parity or age of children at discharge. If mothers insured by the Helsana Group differ from mothers insured elsewhere with regard to risks in pregnancy and delivery, this might affect the findings. We have, however, no evidence that there is a positive or negative risk selection associated with insurance from the Helsana Group.

A strength of the study is that we could take into account costs after hospital discharge for mothers and infants over a period of six months and costs could be retrieved for 97% of all claims relating to the study period. In addition to before/after comparisons, we could also conduct cross-sectional comparisons between the intervention canton and five cantons without a coordinated care offer and triangulate the respective findings. We did not restrict our analysis to healthy full-term children.

## Conclusion

Although the introduction of a coordinated postpartum care model increased the access and use of midwifery home care, there was no cost increase for mothers but a cost reduction for neonates during the first month after hospital discharge. Further research should include a continuous evaluation over extended periods of time, and enable the assessment of both economic as well as health outcomes.
